# Case Report: Surgical repair of mitral prolapse caused by papillary muscle elongation using premeasured artificial chordae: a case series

**DOI:** 10.3389/fcvm.2025.1543876

**Published:** 2025-07-08

**Authors:** Lixi Gan, Oudi Chen, Fanyu Chen, Weiteng Wang, Hongkun Qing, Xuhua Jian

**Affiliations:** ^1^ Department of Cardiac Surgery, Guangdong Provincial People’s Hospital (Guangdong Academy of Medical Sciences), Southern Medical University, Guangzhou, China; ^2^ Department of Cardiovascular Surgery, Fuwai Hospital, National Center for Cardiovascular Diseases, Chinese Academy of Medical Sciences and Peking Union Medical College, Beijing, China; ^3^ Department of Echocardiography, Guangdong Provincial People’s Hospital (Guangdong Academy of Medical Sciences), Southern Medical University, Guangzhou, China

**Keywords:** cardiac valve surgery, mitral valve, mitral valve repair, mitral valve prolapse, papillary muscle abnormalities

## Abstract

Artificial chordae are increasingly used to repair mitral prolapse. The application of premeasured artificial chordae facilitates the creation of chordae with an appropriate length. Herein, we describe our experience of applying premeasured artificial chordae in 14 cases of mitral prolapse caused by elongated papillary muscles, with an emphasis on the importance of restoring the physiological features of sub-valvular structures during mitral valve repair.

## Introduction

Artificial chordae are increasingly used to repair abnormal sub-valvular structures with excellent long-term results (1). The primary challenges of this technique include determining and maintaining correct chordal lengths. Additionally, papillary muscle (PM) variations may influence surgical complexity (2). Von Oppell developed the concept of premeasured loops to allow for the creation of chordae with appropriate lengths (1). This technique can be used to repair mitral valves, especially those that exhibit prolapse due to elongated or ruptured chordae (3). However, few articles have reported the application of premeasured loops in patients with abnormal PMs. Here, we describe our experience of using premeasured artificial chordae in 14 cases of mitral prolapse caused by elongated PMs, highlighting the importance of restoring the physiological features of sub-valvular structures during mitral valve repair.

## Description of the cases

**Figure 1:**
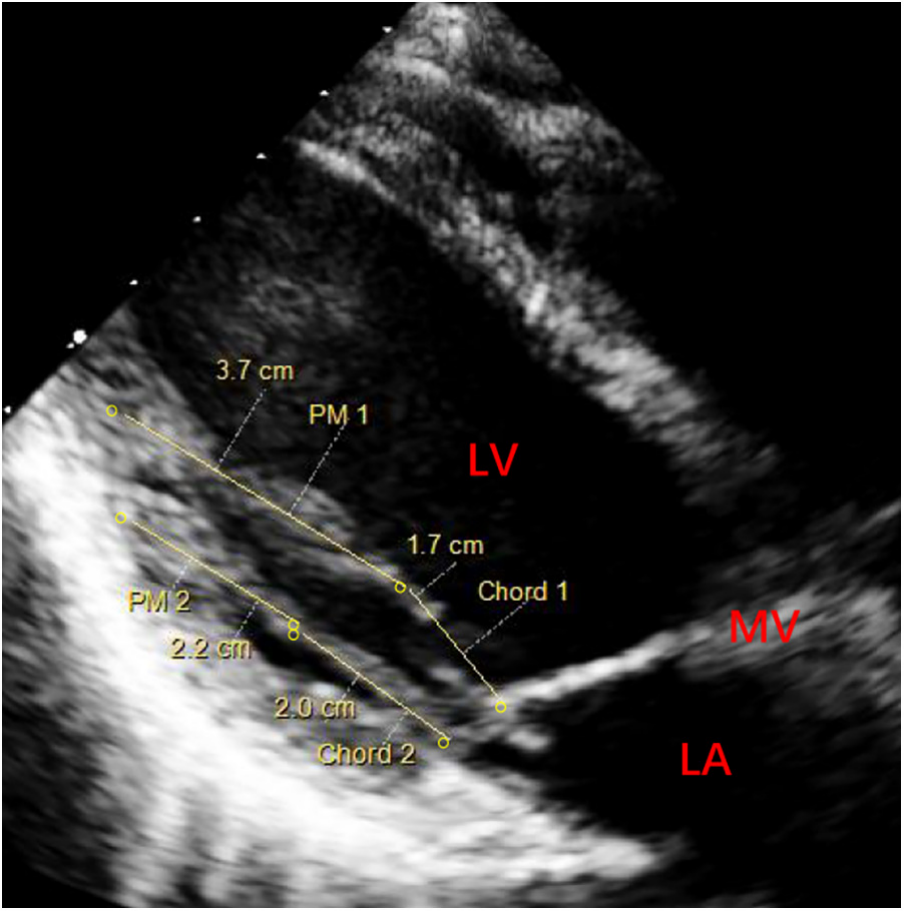
Preoperative echocardiography (parasternal long-axis view) of left ventricular systole for measuring the length of papillary muscles. PM 1 (elongated PM, 3.7 cm) and Chord 1 (the chord of abnormal PM, 1.7 cm). PM 2 (normal PM, 2.2 cm) and Chord 2 (normal chord, 2.0 cm). PM, papillary muscle; MV, mitral valve; LA, left atrium; LV, left ventricle.

We conducted a case series of all patients diagnosed with mitral prolapse caused by PMs elongation through their preoperative echocardiography. According to their echocardiographic images, we measured the length of the chordae and papillary muscles during left ventricular systole (Figure 1). The length of PM was the distance from the endocardial surface at the left ventricular base to the fibrous head of PM. The length of the chord was the distance from the fibrous head of PM to the segment of the leaﬂet. Besides, we ruled out other heart diseases (such as coronary artery disease, congenital heart diseases, hypertrophic obstructive cardiomyopathy, etc.) based on the patient's echocardiography and laboratory findings. The registry collects comprehensive data on baseline patient characteristics, surgical details, in-hospital outcomes, and annual clinical follow-up.

### Operative details

**Figure 2:**
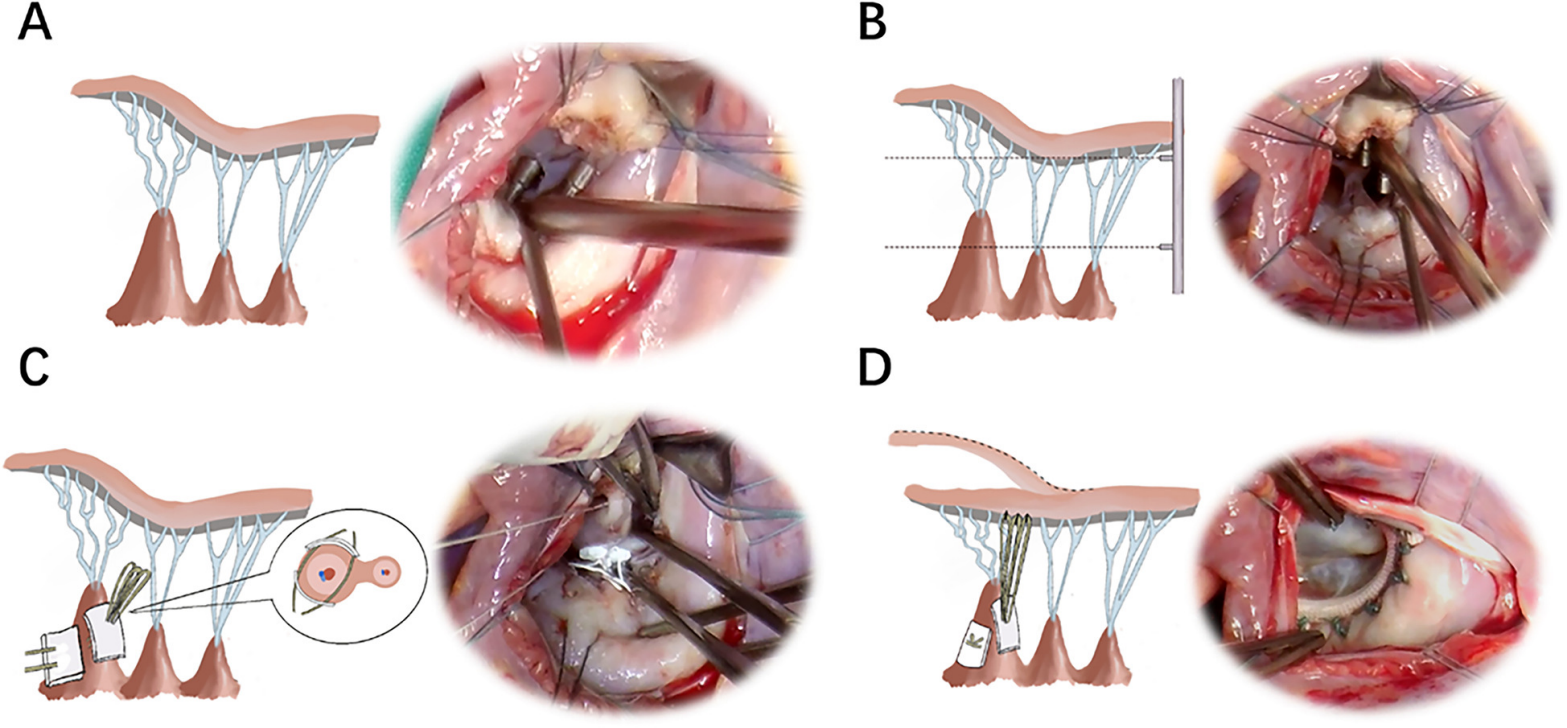
Schematic diagrams and photographs of the operation. **(A)** The mitral leaflet was assessed to confirm the prolapsed position and abnormal sub-valvular apparatus. **(B)** A caliper was used to measure the length of the normal chord and PM. **(C)** The pledget knots were attached to the body of the elongated PM. The sutures were passed through the outer one-third of the PM and carefully tied. **(D)** The artificial chordae were affixed to the leaflet edge. PM, papillary muscle.

The patients underwent midline sternotomy and hypothermic cardiopulmonary bypass. The mitral leaflet was then carefully assessed to confirm the prolapsed position and the presence of abnormal sub-valvular structure(s). In all cases, mitral prolapse was caused by the presence of one PM longer than the others in the same group (Figure 2A). In order to measure the length of a normal chord adjacent to the prolapsed area, a sizing caliper was inserted into the ventricle, with which the tip was placed at the PM insertion of the normal chord (Figure 2B). A single ePTFE CV-4 (4-0 GORE-TEX) was threaded around this caliper and ligated four times to create the artificial chordae loop. For diffuse prolapse, the above process should be repeated based on the number of chords, since the anterior leaflet's free edge can be supported by one single chordal loop for up to 5–10 mm (3).

The loop pledgets were attached to the body of the elongated PM by two sutures, which made the pledgets towards the PM base comparable to the adjacent normal PMs (Figure 2C). To reduce injury to the PM, each suture was carefully threaded through the outer one-third of the PM with a second pledget and gently ligated 10 times (Figure 2C). During this process, a back-handed technique was used to pass the needles, in which the chords arose from the portion of the PM facing the surgeon. Each chordal loop was sequentially attached to the prolapsing segment of the leaﬂet from the ventricular to the atrial side with other ePTFE CV-5 (5-0 GORE-TEX) for reconstructing the artificial chordae (Figure 2D). The loops were attached to the anterior PM for A1 and the lateral half of A2, as well as to the posterior PM for A3 and the medial half of A2 (3). Additionally, an appropriate annular ring was selected to preserve the flexibility of the valve annulus, according to the distance between the two commissures, the surface area of the anterior leaflet, and the coaptation height. Valve function was assessed by injecting saline across the mitral valve under pressure. After completion of de-airing and discontinuation of bypass, valve function was assessed using transesophageal echocardiography.

## Results

**Figure 3:**
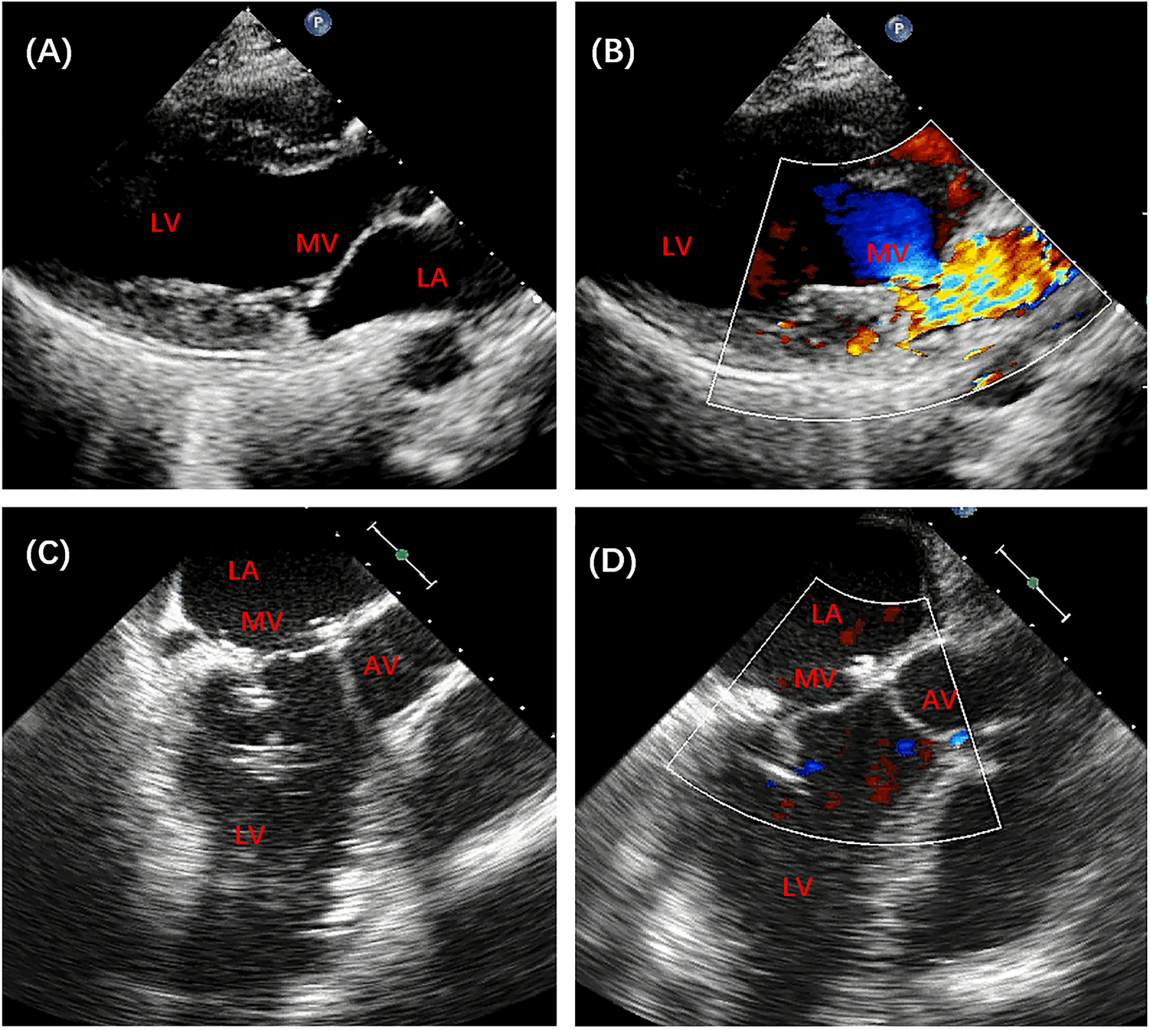
Preoperative and postoperative echocardiography of patient no. 13. **(A,B)** Preoperative echocardiography revealed severe mitral insufficiency. **(C,D)** Postoperative echocardiography showed good mitral valve function. AV, aortic valve; MV, mitral valve; LA, left atrium; LV, left ventricle.

From July 2017 to June 2022, premeasured artificial chordae were used in 14 patients with moderately severe or severe mitral prolapse caused by elongated PMs. The patients' mean age was 32 ± 14.5 years and nine (64.3%) were male, as shown in Table 1. All patients had A2–A3/A3 prolapse of the anterior mitral leaflet segments caused by elongated posteromedial PMs. The mean cardiopulmonary bypass and aortic cross-clamp times were 95 ± 17.2 min and 138 ± 17.5 min. Short-term postoperative echocardiographic images (Figure 3) showed trivial or no valvular regurgitation. Furthermore, the sutured PMs showed no significant signs of ischemia or rupture. All patients were discharged without any adverse events. At their 1–6-year follow-ups, none of the patients reported needing reoperation, and all survived with New York Heart Association class I or II cardiac function.

**Table 1:** The clinical characteristics and follow-up results of 14 patients.

Patient characteristics	Diagnosis	Preoperative echocardiography	Operation	Number of implanted loops	CPB (min)	ACC (min)	Size of the annuloplasty ring	Postoperative echocardiography	Follow-up
48Y, man	Severe mitral insufficiency, NYHA-II	MRA: 7.3 cm^2^, LVEF: 65%, mitral anterior prolapse (A2-A3)	Mitral valve repair, premeasured artificial chordae	3	136	93	30	6 years after surgery MRA: 3.0 cm^2^, LVEF: 62%	Alive at 6 years follow-up with NYHA-II
18Y, woman	Severe mitral insufficiency, NYHA-III	MRA: 8.8 cm^2^, LVEF: 56%, mitral anterior prolapse (A2-A3)	Mitral valve repair, premeasured artificial chordae, plication of posteromedial commissure	3	145	97	28	1 year after surgery MRA: 1.0 cm^2^, LVEF: 70%	Alive at 6 years follow-up with NYHA-I
30Y, woman	Moderately severe mitral insufficiency, NYHA-II	MRA: 7.1 cm^2^, LVEF: 62%, mitral anterior prolapse (A2–A3)	Mitral valve repair, premeasured artificial chordae	3	120	75	26	9 months after surgery MRA: 0.0 cm^2^, LVEF: 60%	Alive at 6 years follow-up with NYHA-I
35Y, man	Moderately severe mitral insufficiency, NYHA-II	MRA: 6.1 cm^2^, LVEF: 66%, mitral anterior prolapse (A3)	Mitral valve repair, premeasured artificial chordae, plication of posteromedial commissure	2	156	119	30	4 years after surgery MRA: 4.2 cm^2^, LVEF: 67%	Alive at 6 years follow-up with NYHA-I
23Y, man	Severe mitral insufficiency, NYHA-II	MRA: 13.8 cm^2^, LVEF: 68%, mitral anterior prolapse (A3)	Mitral valve repair, premeasured artificial chordae	3	128	106	28	5 years after surgery MRA: 1.0 cm^2^, LVEF: 65%	Alive at 6 years follow-up with NYHA-I
27Y, man	Moderately severe mitral insufficiency, NYHA-II	MRA: 6.6 cm^2^, LVEF: 65%, mitral anterior prolapse (A2–A3)	Mitral valve repair, premeasured artificial chordae	3	128	82	30	6 months after surgery MRA: 0.8 cm^2^, LVEF: 66%	Alive at 5 years follow-up with NYHA-I
52Y, man	Severe mitral insufficiency, NYHA-II	MRA:8.3 cm^2^, LVEF: 65%, mitral anterior prolapse (A2–A3)	Mitral valve repair, premeasured artificial chordae, plication of posteromedial commissure	3	166	128	32	1 year after surgery MRA: 0.7 cm^2^, LVEF: 72%	Alive at 4 years follow-up with NYHA-I
58Y, man	Severe mitral insufficiency, NYHA-II	MRA:13.5 cm^2^, LVEF: 61%, mitral anterior prolapse (A2–A3)	Mitral valve repair, premeasured artificial chordae	3	96	74	34	11 months after surgery MRA: 5.9 cm^2^, LVEF: 55%	Alive at 4 years follow-up with NYHA-II
31Y, man	Severe mitral insufficiency, NYHA-II	MRA:16.2 cm^2^, LVEF: 65%, mitral anterior prolapse (A2–A3)	Mitral valve repair, premeasured artificial chordae	2	161	111	26	4 years after surgery MRA: 1.7 cm^2^, LVEF: 60%	Alive at 4 years follow-up with NYHA-I
55Y, man	Severe mitral insufficiency, NYHA-II	MRA:14.4 cm^2^, LVEF: 65%, mitral anterior prolapse (A3)	Mitral valve repair, premeasured artificial chordae	3	153	86	28	4 years after surgery MRA: 1.3 cm^2^, LVEF:62%	Alive at 4 years follow-up with NYHA-II
16Y, woman	Severe mitral insufficiency, NYHA-I	MRA:8.7 cm^2^, LVEF: 67%, mitral anterior prolapse (A2–A3)	Mitral valve repair, premeasured artificial chordae	3	107	75	30	3 years after surgery MRA: 0.0 cm^2^, LVEF: 51%	Alive at 3 years follow-up with NYHA-I
15Y, woman	Severe mitral insufficiency, NYHA-IV	MRA:12.5 cm^2^, LVEF: 66%, mitral anterior prolapse (A2-A3-PC)	Mitral valve repair, premeasured artificial chordae	3	135	90	—	4 years after surgery MRA: 0.0 cm^2^, LVEF: 73%	Alive at 4 years follow-up with NYHA-I
21Y, woman	Severe mitral insufficiency, NYHA-III	MRA:8.4 cm^2^, LVEF: 63%, mitral anterior prolapse (A2–A3-PC)	Mitral valve repair, premeasured artificial chordae	3	149	106	26	2 years after surgery MRA: 1.3 cm^2^, LVEF: 70%	Alive at 2 years follow-up with NYHA-I
26Y, man	Severe mitral insufficiency, NYHA-II	MRA:9.8 cm^2^, LVEF: 70%, mitral anterior prolapse (A2–A3)	Mitral valve repair, premeasured artificial chordae	3	169	115	26	2 years after surgery MRA: 0.7 cm^2^, LVEF: 63%	Alive at 2 years follow-up with NYHA-I

NYHA, New York Heart Association functional class; MRA, jet mitral regurgitant area; LVEF, left ventricular ejection fraction; CPB, cardiopulmonary bypass time; ACC, aortic cross-clamp time.

## Discussion

Since its first description two decades ago (1), the premeasured loops technique has become increasingly popular. Gillinov described and illustrated this surgical technique in detail for repairing mitral prolapse caused by elongated or ruptured chordae (3). However, few articles have reported the application of this technique in patients with abnormal PMs. Here, we introduce our experience of using the premeasured artificial chordae for repairing mitral prolapse caused by elongated PMs.

PMs vary in length and breadth, which may influence the complexity of valve repair surgery (2). Moreover, it is difficult to determine an appropriate chordal length for mitral prolapse caused by elongated PMs. Herein, we describe our experience using premeasured artificial chordae in 14 cases of mitral prolapse caused by elongated PMs, which could be an addition to the surgical indications for this procedure. Methods utilizing premeasured artificial chordae could assist surgeons in determining the loop length and in making chordal replacement less subjective. We adopted a lower attachment position of the loops to maintain the length of the artificial chordae in the case of these special sub-valvular structures (compared to that used for the pledgets, which were attached at the tip of the PMs). During our clinical experience, all cases had anterior leaﬂet prolapse caused by abnormal posteromedial PMs; however, this technique may also be used in cases of posterior leaﬂet prolapse.

PM elongation disrupts the normal mitral valve mechanics by altering the force distribution between the PM and chordae tendineae, leading to asymmetric leaflet tethering and eccentric regurgitation. This distinct pathophysiology presents unique challenges in determining chordal length, as traditional reference points become unreliable when elongated sub-valvular structures are present (2). As with Von Oppell's (1), we demonstrated that premeasured artificial chordae provide reliable repair of sub-valvular structures. In patients with mitral prolapse secondary to PM elongation, the artificial chordae were constructed to match the length of adjacent normal chordae, thereby restoring the sub-valvular ratio and facilitating physiological leaflet movement. These results underscore the importance of restoring the physiological characteristics of sub-valvular structures during mitral valve repair, particularly in cases with elongated PMs or chordae. The premeasured artificial chordae technique offers several distinct advantages over conventional approaches for managing PM elongation. When compared with the standard interrupted neochordae (4), this approach facilitates simultaneous implantation of multiple chordae (mean 2.8 ± 0.4 per case), thereby enabling more efficient correction of diffuse prolapse. In comparison with the running technique (5), this method significantly reduces PM trauma through the elimination of repetitive suture penetration. Compared with the standard loop technique (6), our technique provides more physiological length restoration by utilizing adjacent native chordae as reference, thus maintaining sub-valvular geometry more effectively. These technical advantages are particularly relevant for PM elongation cases where maintaining natural force distribution is paramount.

Although we attached the loops to the body of the PM, we did not observe any tearing, ischemic changes, or adverse clinical/echocardiographic outcomes. One may find answers in the follow-up of other mitral valve repair techniques, such as homograft replacement. Hvass et al. (7) observed that while their procedures on PMs may cause damage, there were no cases of PM rupture in follow-up.

## Conclusion

Premeasured artificial chordae effectively repaired mitral prolapse caused by elongated PMs, demonstrating stable mid-term outcomes. While limited by sample size, this standardized approach shows promise for focal lesions, warranting further validation in complex pathologies.

## Data Availability

The original contributions presented in the study are included in the article/Supplementary Material, further inquiries can be directed to the corresponding author.

## Supplementary material

Supplementary Video S1 Preoperative and postoperative echocardiography of patient no. 13.

Supplementary Video S2 Surgical technique.
